# Negative Aspects of Close Relationships as Risk Factors for Cognitive Aging

**DOI:** 10.1093/aje/kwu236

**Published:** 2014-10-22

**Authors:** Jing Liao, Jenny Head, Meena Kumari, Stephen Stansfeld, Mika Kivimaki, Archana Singh-Manoux, Eric J. Brunner

**Keywords:** aging, cognitive decline, longitudinal studies, social relationships

## Abstract

The extent to which social relationships influence cognitive aging is unclear. In this study, we investigated the association of midlife quality of close relationships with subsequent cognitive decline. Participants in the Whitehall II Study (*n* = 5,873; ages 45–69 years at first cognitive assessment) underwent executive function and memory tests 3 times over a period of 10 years (1997–1999 to 2007–2009). Midlife negative and positive aspects of close relationships were assessed twice using the Close Persons Questionnaire during the 8 years preceding cognitive assessment. Negative aspects of close relationships, but not positive aspects, were associated with accelerated cognitive aging. Participants in the top third of reported negative aspects of close relationships experienced a faster 10-year change in executive function (−0.04 standard deviation, 95% confidence interval: −0.08, −0.01) than those in the bottom third, which was comparable with 1 extra year of cognitive decline for participants aged 60 years after adjustment for sociodemographic and health status. Longitudinal analysis found no evidence of reverse causality. This study highlights the importance of differentiating aspects of social relationships to evaluate their unique associations with cognitive aging.

There is increasing evidence of the impact of social relationships on multiple aspects of health (1–4). The hypothesized neuroprotective effect of social relationships (5–7) is of substantial public health relevance, particularly because they may act as modifiable risk factors for dementia (8–10).

Through provision of reassurance or tangible aid, social relationships bolster individual self-esteem and contribute to well-being (11). However, well-intentioned interpersonal support may elicit an unpleasant social exchange if the recipient finds the relationship intrusive or overcontrolling (12). Negative aspects of social relationships not only affect daily mood but also may adversely influence mental and physical health (13–16), with more potent and longer-lasting associations than positive aspects (17–19).

Equivocal evidence has accumulated regarding social relationships and cognitive aging. For example, Seeman et al. (20) showed that a history of greater negative aspects of social exchanges predicted poor executive function as measured on a single occasion 10 years after baseline. In contrast, other studies found that negative aspects of social relationships were associated with better concurrent cognition but found no evidence of longitudinal associations (21, 22). Concurrent associations between confiding support (described below) and better cognition have been observed (22–24), yet only 1 study has demonstrated this positive association longitudinally (22). Likewise, the effect of practical support (described below) remains unclear, with both adverse (25) and beneficial (26) impacts on cognitive decline. Few studies so far have considered distinct aspects of social relationships simultaneously (20–22). Given that close relationships are likely to involve both negative and positive exchanges (27), it may be more informative to study how they work in tandem (28).

Accordingly, we investigated the impact of midlife negative and positive aspects of close relationships on subsequent cognitive decline up to early old age, using data on the Whitehall II cohort, with 3 assessments of cognitive function spanning 10 years. Further, we scrutinized the results for potential reverse causality of these associations.

## METHODS

### Study population

In the Whitehall II Study, a cohort of 10,308 participants aged 35–55 years (66% male) was recruited from 20 London, United Kingdom-based civil service departments in 1985–1988. At study baseline, all participants underwent clinical health checkups and completed self-administrated questionnaires. Subsequent data collection alternated between postal questionnaires alone and postal questionnaires accompanied by clinical checkups (29). Cognitive tests were introduced to the entire cohort in phase 5 (1997–1999), with repeated measures in phase 7 (2002–2004) and phase 9 (2007–2009). Ethical approval for the Whitehall II Study was obtained from the University College London Medical School Committees on the Ethics of Human Research. All participants were asked to give written informed consent during each phase.

### Close relationships

The Close Persons Questionnaire was used to assess social relationships (30). The questionnaire was introduced halfway through phase 1. Measurements of close relationships were available for phases 2 (1989–1990), 5, 7, and 9. Three subscales were derived from factor analysis (30): “negative aspects of close relationships,” “confiding support,” and “practical support.” Negative aspects of close relationships (4 items; Cronbach's α = 0.65) captured adverse interactions (e.g., producing worries, problems, and stress) and lack of adequate support (e.g., needing more help). Confiding support (7 items; Cronbach's α = 0.86) and practical support (3 items; Cronbach's α = 0.80) reflected positive aspects of support (e.g., giving suggestions, sharing interests, or boosting self-esteem and tangible aid/helping behaviors, respectively). Each item was rated on a 4-point Likert scale, with higher scores indicating greater negative or positive support. To summarize the history of social relationships in midlife, we calculated a cumulative score for quality of close relationships by averaging the phase 2 and phase 5 scores for each support subscale. If a value for either phase was missing, the score from the available phase was imputed as the cumulative score, as there was fairly high correlation between measures (0.54 for confiding support, 0.45 for practical support, and 0.42 for negative aspects of close relationships). Because there was evidence of a nonlinear association in preliminary analyses, the scaled responses were summed and divided into 3 groups based on phase 2 tertile cutpoints.

### Cognitive function

Cognitive tests were administered 3 times over a 10-year follow-up period (in phases 5, 7, and 9) and comprised a test of short-term verbal memory, part I of the Alice Heim 4 test, and a test of verbal fluency. Short-term verbal memory was measured using a 20-word audiotaped list of single- or double-syllable words spoken at 2-second intervals, which participants were required to recall in writing within 2 minutes (31). The Alice Heim 4 part I test, an inductive reasoning test, consists of 65 verbal and numerical items to be completed within 10 minutes (32). This test measures one's ability to identify patterns and infer principles. Two measures of verbal fluency were assessed: phonemic fluency and semantic fluency. Participants were instructed to recall, in writing, as many words beginning with “S” as possible in 1 minute (phonemic fluency) and as many animal names as possible in 1 minute (semantic fluency) (33). A composite score of executive function was created using the Alice Heim 4 part I and verbal fluency tests by first standardizing the raw scores from each test to *z* scores (mean = 0; standard deviation (SD), 1) based on the phase 5 mean value and SD; then these *z* scores were averaged to obtain the executive function score.

### Covariates

Sociodemographic variables included were age, year of birth, sex, and ethnicity (white/nonwhite). Two measures of socioeconomic status were used: educational attainment (i.e., no formal education, up to secondary education, and university or higher degree) and British civil service employment grade (i.e., clerical or support grades (low), professional or executive grades (medium), and administrative grades (high)).

Prevalent chronic disease was defined as having coronary heart disease, stroke, diabetes, or depressive symptoms. Coronary heart disease prevalence was ascertained according to clinically verified events, including myocardial infarction and definite angina. Information on stroke was obtained from self-reports of a physician's diagnosis. Diabetes was assessed on the basis of the World Health Organization diagnostic criteria for diabetes: a fasting plasma glucose concentration of ≥7.0 mmol/L (126 mg/dL) or a 2-hour plasma glucose concentration of ≥11.1 mmol/L (200 mg/dL), reported physician-diagnosed diabetes, or use of diabetes medication (34). History of depressive symptoms was defined as a General Health Questionnaire score greater than 4.

### Statistical analysis

Characteristics of participants across different categories of negative aspects of social relationships were assessed by χ^2^ test for categorical variables and by analysis of variance for continuous variables. Multilevel modeling was used to investigate the impact of midlife close relationships on cognitive decline from middle age to early old age (approximately 55–65 years). Trajectories of cognitive decline were modeled using cognitive data from phases 5, 7, and 9. Core terms included in the model were age (centered at age 60 years and divided by 10 to obtain change over 10 years), age squared, year of birth (centered at 1940 to adjust for cohort effects (35, 36)), sex, ethnicity, history of close relationships, and an interaction term for the interaction of each covariate with age. (The random effect of age squared cannot be computed. Given that interactions with the quadratic age slope were not significant, we simplified the models by using interaction with linear age slope only.) Three-way interaction terms for interactions between sex, age, and close relationships suggested that there were no sex differences in the associations between close relationships and cognitive decline (*P*'s = 0.27–0.94). Therefore, men and women were combined in the analysis, with adjustment for sex. The key interaction term for interaction between close relationships and age tested whether close relationships were related to cognitive decline. The intercept and slope were fitted as random effects, allowing for variation in the intercept and rate of cognitive decline. Next, socioeconomic status (model 2) and prevalent chronic disease (model 3), along with their interactions with age, were added to the model to adjust for their effects on the association between close relationships and cognitive decline. The magnitudes of the associations between close relationships and cognitive decline were compared with the longitudinal aging effect on cognitive decline estimated from model 3. The formula used was (difference in 10-year decline in cognitive function between the highest and lowest thirds of the close relationships indicator)/(cognitive decline over 1 year for persons aged 60 years).

The relative importance of negative and positive aspects of close relationships was examined via simultaneously entering all markers of social relationships into model 3. We carried out sensitivity analysis by excluding participants with Mini-Mental State Examination scores less than 23 (possible dementia cases) during the 10-year follow-up period. Finally, to assess reverse causality, we used phase 5 cognitive scores (entered as continuous variables) to predict changes in close relationships from phase 5 to phase 9 using multilevel models (*n* = 5,958, of whom 98% were participants included in the main analysis). This analysis tested whether participants with initially better cognition would encounter less negative and more positive social exchanges.

All analyses were performed with STATA SE, version 13.1 (StataCorp LP, College Station, Texas). A *P* value below 0.05 (2-sided) was considered statistically significant.

## RESULTS

Of the 10,308 persons who participated at the inception of the Whitehall II Study (1985–1988), 306 had died and 752 had withdrawn before the start of cognitive data collection in phase 5 (1997–1999). Among the 9,250 participants remaining in the cohort, 7,495 completed at least 1 of the 3 cognitive tests over a period of 10 years. Analyses were based on 5,873 participants who completed 1 or more cognition tests and had data on negative aspects of close relationships and other covariates. Of these individuals, 4,317 (73.5%) had complete data for all 3 phases, 957 (16.3%) for 2 phases, and 599 (10.2%) for only 1 phase. The levels of close relationships were similar between persons with complete data for all 3 phases and those with 1 or 2 missing cognitive tests (*P*'s = 018–0.69). Compared with those remaining in the cohort but not eligible for analysis (*n* = 3,377), included participants tended to be younger (55.7 years in phase 5 vs. 56.8 years in phase 5; *P* < 0.0001), male (71.6% vs. 61.5%; *P* < 0.001), and better educated (university degree or beyond: 41.7% vs. 28.9%; *P* < 0.001) and had higher occupational grades (administrative level: 43.9% vs. 33.5%; *P* < 0.001).

Table 1 gives the characteristics of participants according to history of negative aspects of close relationships. Participants who were younger and participants who reported less confiding support or less practical support were more likely to report negative aspects of close relationships. Reporting negative aspects of close relationships was also related to nonwhite ethnicity and higher educational level. The prevalences of diabetes and depressive symptoms were highest among those who reported high cumulative negative aspects of close relationships.

**Table 1.** Characteristics (%)^a^ of Participants in Phase 5 (1997–1999) According to History of Negative Aspects of Close Relationships (1989–1990 to 1997–1999), Whitehall II Study, United Kingdom

Characteristic	Cumulative Level of Negative Aspects of Close Relationships^b^			*P* for Heterogeneity
	Low	Medium	High	
	(*n* = 1,570)	(*n* = 2,865)	(*n* = 1,438)	
Age, years^c^	56.7 (6.1)	55.3 (6.0)	55.3 (6.0)	<0.0001
Male sex	69.8	72.4	72.2	0.17
White ethnicity	96.1	93.8	85.1	<0.001
High occupational position^d^	42.3	45.8	42.1	0.01
University degree or higher	35.9	43.2	45.1	<0.001
Prevalent chronic disease				
Coronary heart disease	5.7	5.8	7.0	0.28
Stroke	0.4	0.4	0.6	0.59
Diabetes	6.1	5.0	7.3	0.004
Depressive symptoms	11.0	21.2	34.1	<0.01
High level of confiding support^b^	17.6	9.3	5.7	<0.01
High level of practical support^b^	17.5	15.7	14.0	0.002

^a^ Numbers are percentages unless otherwise stated.

^b^ Cutoff points: for negative aspects of close relationships—low, <1.2; medium, <3.5; for confiding support—low, <13.5; medium, <18.5; for practical support—low, <4.8; medium, <7.2.

^c^ Values are mean (standard deviation).

^d^ A British civil service employment grade on the administrative level.

**Figure 1.**
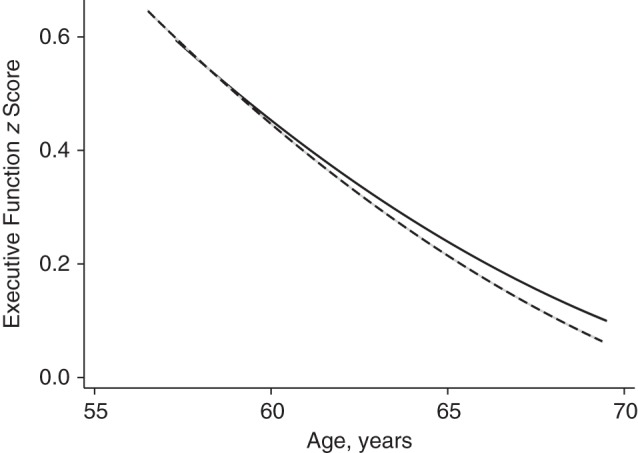
Predicted trajectory of executive function with age according to cumulative level of negative aspects of close relationships, Whitehall II Study, United Kingdom, 1997–1999 to 2007–2009. The solid line represents a low cumulative level of negative aspects; the dashed line represents medium and high cumulative levels of negative aspects (*P* for difference = 0.01). The graph shows the predicted trajectory for white male Whitehall II participants born in 1940 who were in the highest employment grade (administrative) and had a university education and no prevalent chronic diseases in phase 5 (1997–1999).

Table 2 shows the associations between cumulative negative aspects of close relationships and cognitive decline over 10 years (phases 5, 7, and 9). Participants reporting a high level of negative aspects of close relationships showed the fastest decline in executive function, with a 10-year change that was accelerated by −0.05 SD (95% confidence interval (CI): −0.09, −0.01) in comparison with those reporting low negative aspects of close relationships (model 1). Adjustment for socioeconomic status and prevalent chronic disease together (model 3) attenuated this association by 20%, yet a greater decline (−0.04 SD, 95% CI: −0.08, −0.01) was still evident among persons with higher negative aspects of close relationships (Figure 1). The estimated 10-year change in executive function for participants aged 60 years was −0.40 SD (95% CI: −0.42, −0.39) (results not shown). Thus, the −0.04-SD difference in 10-year decline in executive function between the highest and lowest thirds of negative aspects of close relationships corresponds to 1 extra year of decline in executive function for participants aged 60 years. In contrast, the rate of cognitive decline did not differ by positive aspects of social relationships in either the confiding support group or the practical support group (Appendix Table 1).

**Table 2.** Association Between History of Negative Aspects of Close Relationships (1989–1990 to 1997–1999) and 10-Year Cognitive Decline (1997–1999 to 2007–2009), Whitehall II Study, United Kingdom

Cognitive Function, by Level of Negative Aspects of Close Relationships	Model 1^a^		Model 2^b^		Model 3^c^	
	β^d^	95% CI	β^d^	95% CI	β^d^	95% CI
Standardized executive function						
Low	0	Referent	0	Referent	0	Referent
Medium	−0.04	−0.07, −0.02	−0.04	−0.07, −0.01	−0.04	−0.07, −0.02
High	−0.05	−0.09, −0.01	−0.04	−0.08, −0.01	−0.04	−0.08, −0.01
*P* for interaction^e^	0.02		0.03		0.05	
Standardized memory						
Low	0	Referent	0	Referent	0	Referent
Medium	−0.04	−0.09, 0.01	−0.03	−0.08, 0.02	−0.03	−0.08, 0.02
High	−0.02	−0.08, 0.04	−0.01	−0.06, 0.05	−0.01	−0.06, 0.06
*P* for interaction^e^	0.33		0.32		0.29	

Abbreviation: CI, confidence interval.

^a^ Model 1: adjusted for cohort, sex, and ethnicity.

^b^ Model 2: adjusted for model 1 variables + socioeconomic status (education and employment grade).

^c^ Model 3: adjusted for model 2 variables + prevalent chronic disease (coronary heart disease, stroke, diabetes, and depressive symptoms).

^d^ The regression coefficient (β) indicates the difference in 10-year cognitive decline relative to each reference group.

^e^ The interaction term tested whether cognitive decline (fixed effect) differed across the 3 categories of negative aspects of close relationships (i.e., low, medium, and high).

When all 3 scales of close relationships were entered into the fully adjusted model, the significant association between negative aspects of close relationships and cognitive aging remained. The difference in 10-year decline in executive function between the highest third and lowest third of negative aspects of close relationships was −0.04 SD (95% CI: −0.07, −0.01). Similar results were obtained after excluding participants with Mini-Mental State Examination scores less than 23 during follow-up (*n* = 57). We evaluated the associations between cognitive function and subsequent changes in the quality of close relationships to investigate the possibility of reverse causation (Table 3). Cognitive function in phase 5 was not associated with either the intercept (for executive function, *P*'s = 0.37–0.80; for memory, *P*'s = 0.07–0.72) or 10-year change in close relationships (for executive function × age, *P*'s = 0.34–0.87; for memory × age, *P*'s = 0.16–0.92).

**Table 3.** Association Between Phase 5 Cognitive Function (1997–1999) and Changes in the Quality of Close Relationships (1997–1999 to 2007–2009), Whitehall II Study, United Kingdom

Cognitive Function	Negative Aspects of Close Relationships			Confiding Support			Practical Support		
	β^a^	95% CI	*P* Value	β^a^	95% CI	*P* Value	β^a^	95% CI	*P* Value
Executive function (*I*)	−0.01	−0.06, 0.04	0.66	0.06	−0.07, 0.18	0.37	0.01	−0.06, 0.08	0.80
Executive function × age (*S*)	−0.03	−0.08, 0.03	0.34	0.05	−0.06, 0.16	0.36	−0.01	−0.08, 0.06	0.87
Memory (*I*)	−0.04	−0.08, 0.00	0.07	−0.02	−0.12, 0.08	0.72	0.02	−0.03, 0.08	0.40
Memory × age (*S*)	0.01	−0.04, 0.05	0.78	0.00	−0.10, 0.09	0.92	0.04	−0.02, 0.10	0.16

Abbreviations: CI, confidence interval.

^a^ The regression coefficient (β) indicates the effect of a 1–standard deviation difference in cognitive score in relation to the intercept (*I*) or the 10-year slope (*S*) of close relationships. All models adjusted for cohort, sex, ethnicity, education, and employment grade.

## DISCUSSION

This study showed that participants reporting higher cumulative levels of negative aspects of close relationships over the course of midlife experienced accelerated declines in executive function from middle age to early old age. These associations were attenuated but remained statistically significant after adjustment for sociodemographic characteristics and health status. The findings did not appear to be the product of reverse causality.

Our study extends previous evidence on the association between negative aspects of close relationships and cognitive aging. Seeman et al. (20) showed that social conflicts were related to lower executive function but not to episodic memory, which were assessed 10 years after measurement of social relationships. Our longitudinal investigation highlights the need to assess the continuities of cognitive status that were not discerned from cross-sectional studies, which are also subject to biases related to reverse causality (21, 22, 37). In view of our findings that phase 5 cognitive ability did not significantly influence subsequent close relationship transitions, this study largely ruled out reverse causality and demonstrated an association of negative aspects of close relationships with cognitive aging.

A salutary impact of positive social relationships on cognitive aging was not found in the current study, as has been found in some studies (21, 23, 24) but not others (20, 22, 26). Given that the Whitehall II cohort was a relatively healthy sample of adults in early old age, the experience of less confiding support or practical support may not necessarily influence cognitive decline at this stage. Receipt of more practical support may instead indicate a need for additional assistance, signifying poorer health, as well as foster dependency and erode self-efficacy (25, 38, 39).

Taken together, the results suggest that negative aspects of close relationships, but not positive aspects, are associated with cognitive aging, consistent with previous evidence on other health outcomes (14–16, 18, 19). This asymmetrical impact could lie in disproportionate rumination on negative social encounters (40), which evokes a stronger emotional reaction (17, 41) and becomes a source of chronic strain (42, 43). The estimated difference in the rate of cognitive decline due to negative aspects of close relationships was −0.04 SD (top third vs. bottom third), which is comparable with 1 extra year of decline in executive function for participants aged 60 years. The modest magnitude of the difference is consistent with findings from other longitudinal studies (20, 44, 45).

The main strengths of this study were its large size, low attrition, and multiple and detailed measurements of cognitive function and social relationships. Repeat measures allowed the use of longitudinal analysis to investigate the impact of history of social relationships on cognitive aging trajectories and vice versa. This enabled us to examine the potential for reverse causality and preclude it as an explanation for the associations observed.

Several limitations of the study should be considered. First, the measures of social relationships were self-reported, so the information may have been influenced by respondents' personality traits (30). Subjective experience, however, reflects individual interpretation of the social environment and has been shown to modify health behaviors (43, 46). These self-rated measures, derived from a well-validated questionnaire, are relevant indictors of social relationships that have established associations with various health outcomes (16, 47, 48). Nevertheless, the measures of perceived support refer to the closest person only; thus, we were unable to investigate the impact of social relationships in a more extended social network (49). Second, the Whitehall II cohort is comprised predominantly of white-collar civil servants, such that the results may not be representative of the general population. Further, in line with those in other longitudinal cohorts, respondents in our cohort were healthier than persons who dropped out of the study or died (50). In our analysis sample, the quality of close relationships was not associated with nonresponse in phase 5. The prevalences of high negative aspects of close relationships were similar among persons who withdrew from the study and those who responded (31.5% vs. 31.2%; *P* = 0.36). Further, none of the 3 close relationship measures in phase 5 differed between persons with complete data and those with 1 or 2 missing cognitive tests over the follow-up period. Nevertheless, it is possible that participants who took the cognitive tests were not a random subsample and that the range of cognitive decline in our study may have been limited (51). Such nonrandom dropout may reduce statistical power to detect an association between close relationships and cognitive aging. Last, we studied only midlife social relationships as potential risk factors for subsequent cognitive decline, with the intention of clarifying temporality. This approach overlooks the dynamic nature of social relationships in later adulthood (52). Further analysis is required to estimate the impact of changes in social relationships from middle age to old age on cognitive aging.

Our longitudinal population-scale study emphasizes the importance of distinguishing between different aspects of close relationships in relation to cognitive aging. We show that negative aspects of close relationships in midlife are adversely related to the rate of cognitive decline. Though no significant association of confiding support or practical support with cognitive decline was found in the current analysis, our study does not preclude the operation of such protective processes in later life. Our findings suggest that it may be especially valuable to reduce negative social encounters and foster protective relationships in facing the challenges of aging.

## Appendix

**Appendix Table 1.** Association Between History of Positive Aspects of Close Relationships (1989–1990 to 1997–1999) and 10-Year Cognitive Decline (1997–1999 to 2007–2009), Whitehall II Study, United Kingdom

Cognitive Function, by Level of Positive Aspects of Close Relationships	Model 1^a^		Model 2^b^		Model 3^c^	
	β^d^	95% CI	β^d^	95% CI	β^d^	95% CI
Standardized executive function						
Confiding support						
Low	0	Referent	0	Referent	0	Referent
Medium	−0.02	−0.05, 0.01	−0.02	−0.05, 0.01	−0.02	−0.06, 0.05
High	−0.03	−0.07, 0.02	−0.02	−0.07, 0.03	−0.02	−0.08, 0.04
Practical support						
Low	0	Referent	0	Referent	0	Referent
Medium	−0.02	−0.05, 0.01	−0.02	−0.05, 0.01	−0.02	−0.05, 0.01
High	−0.04	−0.08, 0.01	−0.03	−0.07, 0.01	−0.03	−0.07, 0.01
Standardized memory						
Confiding support						
Low	0	Referent	0	Referent	0	Referent
Medium	−0.03	−0.07, 0.01	−0.03	−0.07, 0.02	−0.02	−0.07, 0.02
High	0.03	−0.04, 0.10	0.03	−0.04, 0.10	0.03	−0.04, 0.10
Practical support						
Low	0	Referent	0	Referent	0	Referent
Medium	−0.03	−0.07, 0.02	−0.02	−0.07, 0.02	−0.02	−0.07, 0.02
High	−0.03	−0.09, 0.04	−0.01	−0.07, 0.05	−0.01	−0.07, 0.05

Abbreviation: CI, confidence interval.

^a^ Model 1: adjusted for cohort, sex, and ethnicity.

^b^ Model 2: adjusted for model 1 variables + socioeconomic status (education and employment grade).

^c^ Model 3: adjusted for model 2 variables + prevalent chronic disease (coronary heart disease, stroke, diabetes, and depressive symptoms).

^d^ The regression coefficient (β) indicates the difference in 10-year cognitive decline relative to each reference group.
